# CD169^+^ macrophages in lymph node and spleen critically depend on dual RANK and LTbetaR signaling

**DOI:** 10.1073/pnas.2108540119

**Published:** 2022-01-14

**Authors:** Abdouramane Camara, Alice C. Lavanant, Jun Abe, Henri Lee Desforges, Yannick O. Alexandre, Erika Girardi, Zinaida Igamberdieva, Kenichi Asano, Masato Tanaka, Thomas Hehlgans, Klaus Pfeffer, Sébastien Pfeffer, Scott N. Mueller, Jens V. Stein, Christopher G. Mueller

**Affiliations:** ^a^CNRS UPR 3572, University of Strasbourg, Strasbourg 67000, France;; ^b^Department of Oncology, Microbiology and Immunology, University of Fribourg, Fribourg 1700, Switzerland;; ^c^Department of Microbiology and Immunology, The University of Melbourne, The Peter Doherty Institute for Infection and Immunity, Melbourne, Victoria 3000, Australia;; ^d^CNRS Architecture et Réactivité de l’ARN, UPR9002, University of Strasbourg, Strasbourg 67000, France;; ^e^Laboratory of Immune Regulation, School of Life Science, Tokyo University of Pharmacy and Life Sciences, Tokyo 192-0392, Japan;; ^f^Institute of Immunology, Regensburg Center for Interventional Immunology, University Medical Center of Regensburg, Regensburg 93000, Germany;; ^g^Institute of Medical Microbiology and Hospital Hygiene, Heinrich Heine University Düsseldorf, Düsseldorf 40000, Germany

**Keywords:** macrophages, RANK, lymphotoxin, lymph node, spleen

## Abstract

CD169^+^ macrophages reside in lymph node (LN) and spleen and play an important role in the immune defense against pathogens. As resident macrophages, they are responsive to environmental cues to shape their tissue-specific identity. We have previously shown that LN CD169^+^ macrophages require RANKL for formation of their niche and their differentiation. Here, we demonstrate that they are also dependent on direct lymphotoxin beta (LTβ) receptor (R) signaling. In the absence or the reduced expression of either RANK or LTβR, their differentiation is perturbed, generating myeloid cells expressing SIGN-R1 in LNs. Conditions of combined haploinsufficiencies of RANK and LTβR revealed that both receptors contribute equally to LN CD169^+^ macrophage differentiation. In the spleen, the *Cd169*-directed ablation of either receptor results in a selective loss of marginal metallophilic macrophages (MMMs). Using a RANKL reporter mouse, we identify splenic marginal zone stromal cells as a source of RANKL and demonstrate that it participates in MMM differentiation. The loss of MMMs had no effect on the splenic B cell compartments but compromised viral capture and the expansion of virus-specific CD8^+^ T cells. Taken together, the data provide evidence that CD169^+^ macrophage differentiation in LN and spleen requires dual signals from LTβR and RANK with implications for the immune response.

## Significance

The CD169^+^ macrophages that play an important role in the fight against infections and cancer are receptive to environmental signals for their differentiation. We show that lymph node and splenic CD169^+^ macrophages require both LTβR and RANK signaling since the conditional deficiency of either receptor results in their disappearance. Using a reporter mouse, we observe RANKL expression by a splenic mesenchymal cell subset and show that it participates in CD169^+^ macrophage differentiation. Their absence leads to a reduced viral capture and a greatly attenuated virus-specific CD8^+^ T cell expansion. Thus, tight control mechanisms operate for the precise positioning of these macrophages at sites where numerous immune-stimulatory forces converge.

CD169^+^ macrophages are strategically localized at the lymphatic sinuses of lymph nodes (LNs) and the marginal zone of the spleen, where they capture lymph- and blood-borne antigens, respectively (1). These macrophages reside close to B cells and mesenchymal stromal cells. B cells are a constitutive source of lymphotoxin (LT) α and LTβ that bind to the LTβ receptor (R) as LTα_1_β_2_ heterotrimer (2). Lack of B cells and unconditional or B cell–specific ablation of LTα or LTβ lead to loss of CD169^+^ macrophages in LNs and the spleen (3–6). Conversely, B cell–specific overexpression of LTαβ results in an increase of their numbers (7). Furthermore, administration of the decoy fusion protein LTβR-Fc or LTβR inactivation negatively affects their presence in both organs (3, 8, 9). However, although the myeloid cell lineage has been shown to express LTβR (10–12), it remains unclear whether the dependency on LTβR signaling is direct or implies an intermediate cell partner such as the adjacent stromal cells (9, 13, 14).

We have recently shown that receptor activator of NF-κB ligand (RANKL) from marginal zone reticular cells (MRCs) regulates the differentiation of CD169^+^ macrophages in the LN (15). Like LTα and LTβ, RANKL is a member of the TNF superfamily and binds to the signaling receptor RANK (16). Stromal RANKL activates the lymphatic endothelial cells to form a cellular niche for macrophages and directly stimulates their differentiation into the CD169^+^ macrophages of the subcapsular sinus (subcapsular sinus macrophages, SSMs). However, a role of stromal RANKL for the splenic CD169^+^ macrophages has not been addressed. LTαβ and RANKL share many similarities in their biological functions. They are both indispensable for the organogenesis of secondary lymphoid organs (17, 18), are involved in the organogenesis of the thymus (19), and contribute to the formation of the intestinal microfold cells (20). However, RANKL stands out for its role in osteoclastogenesis (16), while LTαβ regulates the production of homeostatic chemokines and the differentiation of follicular dendritic cells (2).

In the context of partially overlapping functions, we scrutinized the implication of the RANK–RANKL and LTβR–LTαβ axes in the differentiation of LN and splenic CD169^+^ macrophages. Using *Cd169*-directed conditional deficiency of RANK or LTβR, we report that direct RANK and LTβR signals are required for their differentiation in the LN and the spleen. In the absence of the receptors, LN CD169^+^ macrophages were replaced by myeloid cells phenotypically similar to the SIGN-R1^+^ medullary sinus macrophages. Deficiency of one copy of either *Rank* or *Ltbr* alleles sufficed for a prominent decrease in macrophage numbers, but the heterozygous deletion of both genes had a compound effect. Altered macrophage differentiation had a negative impact on lymph-borne antigen transport to B cells. In the spleen, *Cd169*-directed RANK or LTβR deficiency elicited a selective loss of the CD169^+^ MMMs. By the use of a RANKL reporter mouse together with RT-qPCR of sorted splenic stromal subsets, we identified CCL19^+^ splenic MRCs as a source of RANKL and demonstrated in *Ccl19-cre* RANKL-deficient mice that stromal RANKL participates in MMM differentiation. Their specific loss had no effect on the marginal zone B cell compartment but compromised viral capture and the formation of the virus-specific CD8^+^ T cell response. Taken together, the data provide evidence that CD169^+^ macrophage differentiation is dependent on the dual signals emanating from LTβR and RANK, with implications for the immune response to lymph- and blood-borne pathogens.

## Results

### Requirement of Direct LTβR and RANK Signaling for Differentiation of SSMs.

**Fig. 1.**
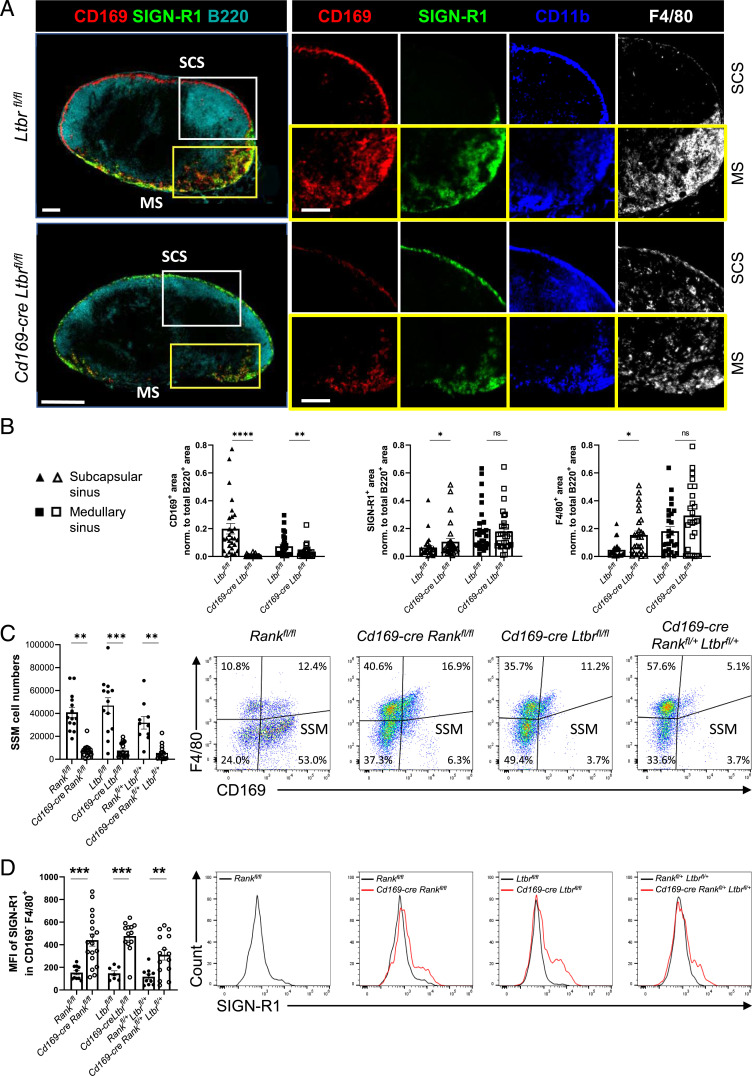
Deficiency in LTβR results in loss of CD169^+^ SSMs. (*A*) Wide-field immunofluorescence microscopic images for CD169, SIGN-R1, CD11b, F4/80, and B220 of inguinal LN sections from *Ltbr^fl/fl^* and *Cd169-cre Ltbr^fl/fl^* mice. White-framed images are higher magnification of the subcapsular sinus (SCS), while yellow-framed images are from the medullary area (MS). Scale bars, 200 µm. (*B*) Quantification of the area of CD169, SIGN-R1, and F4/80 staining in the SCS and MS normalized to total B220^+^ area. Shown are the mean and individual data points from auricular and brachial LNs. Statistical significance (Mann-Whitney test); *P* < 0.005; ns, not significant; error bar, SEM. (*C*) Flow cytometry analysis of SSMs of inguinal and brachial LNs pregated as live CD11b^+^ CD11c^+^ MHC-II^Int^ cells in mice of the indicated genotype. The proportion of the cells in the quadrants is indicated. The graph depicts the mean absolute SSM numbers with data points for each LN. Statistical significance (Kruskal-Wallis test); **P < 0.002, ***P < 0.001; error bar, SEM. (*D*) Mean fluorescence intensity (MFI) of SIGN-R1 in F4/80^+^ CD169^−^ macrophages. Histograms depict representative SIGN-R1 expression for each mutant (red) and control mouse (black). Graph shows the MFI with individual data points for inguinal and brachial LNs. Statistical significance (Kruskal-Wallis test); **P < 0.002; ns, nonsignificant; error bar, SEM.

We have previously shown in *Cd169-cre Rank^fl/fl^* mice that RANK signaling in SSMs is required for their differentiation (15). A replacement of SSMs by CD11b^+^ cells with low expression of CD169 but higher levels of specific ICAM-3–grabbing nonintegrin related 1 (SIGN-R1) is also seen in mice lacking B cells or after administration of LTβR-Fc (3, 7). SIGN-R1 is a marker for LN medullary sinus macrophages and splenic marginal zone macrophages (21, 22). Of note, in the LN, the interfollicular areas that are devoid of B cells lacked CD169^+^ SSMs despite uninterrupted RANKL expression along the subcapsular sinusoids (*SI Appendix*, Fig. S1*A*). Taken together, the data suggest that both RANKL and LTαβ expressed by MRCs and B cells, respectively, are required for SSM differentiation. To address this question directly, we generated *Cd169-cre Ltbr^fl/fl^* mice. Immunofluorescence analysis of LNs of conditionally LTβR-deficient and control mice revealed the presence of CD11b^+^ cells expressing SIGN-R1 and F4/80 within the subcapsular sinus, while CD169 expression was reduced (Fig. 1*A*). Quantification corroborated the increase of SIGN-R1 and F4/80 in the subcapsular zone and the loss of CD169 in subcapsular and medullary zones (Fig. 1*B*; *SI Appendix*, Fig. S1*B*). To extend these analyses, we performed flow cytometry of the LN macrophage population in mice with conditional deficiency in *Ltbr* and *Rank* using a gating strategy to distinguish between the different subsets (*SI Appendix*, Fig. S1*C*) (15). Compared to *Rank^fl/fl^* or *Ltbr^fl/fl^* littermate controls, there was a prominent loss of the CD169^+^ F4/80^−^ SSMs in both *Cd169-cre Rank^fl/fl^* and *Cd169-cre Ltbr^fl/fl^* mice (Fig. 1*C*). To assess further the requirement of both RANK and LTβR signaling for SSM differentiation, we generated mice with the deletion of one floxed allele of *Rank* or of *Ltbr* (*Cd169-cre Rank^fl/+^* and *Cd169-cre Ltbr^fl/+^*) and with deletions of one copy of both genes (*Cd169-cre Rank^fl/+^ Ltbr^fl/+^*). The flow cytometry showed a prominent but incomplete loss of SSMs in *Cd169-cre Rank^fI/+^* and in *Cd169-cre Ltbr^fl/+^* mice (*SI Appendix*, Fig. S1*D*); however, the concurrent ablation of one copy of both genes resulted in a reduction of SSM numbers comparable to the homozygous *Rank* or *Ltbr* gene deletion (Fig. 1*C*). In addition to the loss of the CD169^+^ F4/80^−^ SSMs, there was an increase in the F4/80^+^ CD169^−^ population. Staining for SIGN-R1^+^ uncovered that these macrophages expressed SIGN-R1 at higher levels (Fig. 1*D*). Therefore, SSM differentiation strongly depends on direct signaling of both RANK and LTβR receptors.

**Fig. 2.**
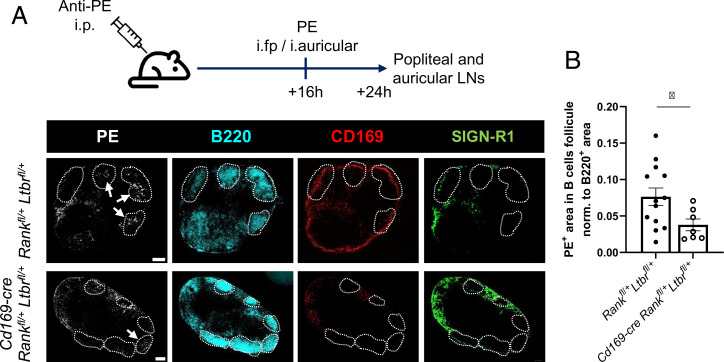
Impaired antigen transport in LNs of compound heterozygous mice. (*A*) Experimental design and representative sections of auricular LNs imaged for PE, B220, CD169, and SIGN-R1. B cell follicles are outlined, and PE accumulated in B cell follicles is pointed out. (*B*) Graph quantifies the PE^+^ area within B cell follicles for *Cd169-cre Rank^fl/+^ Ltbr^fl/+^* and control mice normalized to B220^+^ area per image. Shown are the mean with individual data points of auricular and popliteal LNs. Statistical significance (Mann-Whitney test); *P < 0.05; error bar, SEM (scale bars, 200 μm).

To assess the functional implication, we assessed the transport of particulate antigen from the lymph to B cell follicles, a process relying on SSMs (7, 23). Control and *Cd169-cre Rank^fl/+^ Ltbr^fl/+^* mice received rabbit anti-phycoerythrin (PE) IgG before administration of PE into footpads and ears. The draining popliteal and auricular LNs were imaged by immunofluorescence for localization of PE. We found a significantly reduced PE signal within the B cell follicles in *Cd169-cre Rank^fl/+^ Ltbr^fl/+^* mice compared to littermate controls (Fig. 2 *A* and *B*). We next addressed the question of whether altered SSM differentiation could affect the cell origin and assessed the occupancy of the niche by adoptive bone marrow transfers. We transferred wild-type bone marrow from CD45.1 congenic mice to neonatal nonirradiated control and *Cd169-cre Ltbr^fl/fl^* mice, expecting a postnatal wave of precursor recruitment (24) and thus avoiding irradiation-related events. The adoptive transfer resulted in a low but equal replacement of all macrophage populations, except for a significant increase in CD45.1^+^ CD169^+^ SSMs in LTβR-deficient mice (*SI Appendix*, Fig. S2 *A* and *B*). This supports the conclusion that the niche is not fully occupied by SIGN-R1^+^ CD169^−^ SSMs and/or that their turnover is faster, resulting in more rapid replacement by bone marrow–derived precursors. This suggests that the SSMs in mutant mice partially derive from cells other than embryonic macrophages, such as bone marrow–derived monocytes. Taken together, as a consequence of absent or insufficient receptor expression, the recruited precursors would differentiate into macrophages that differ in phenotype and function from bona fide SSMs.

### Splenic MMMs Depend on RANK and LTβR Signaling.

**Fig. 3.**
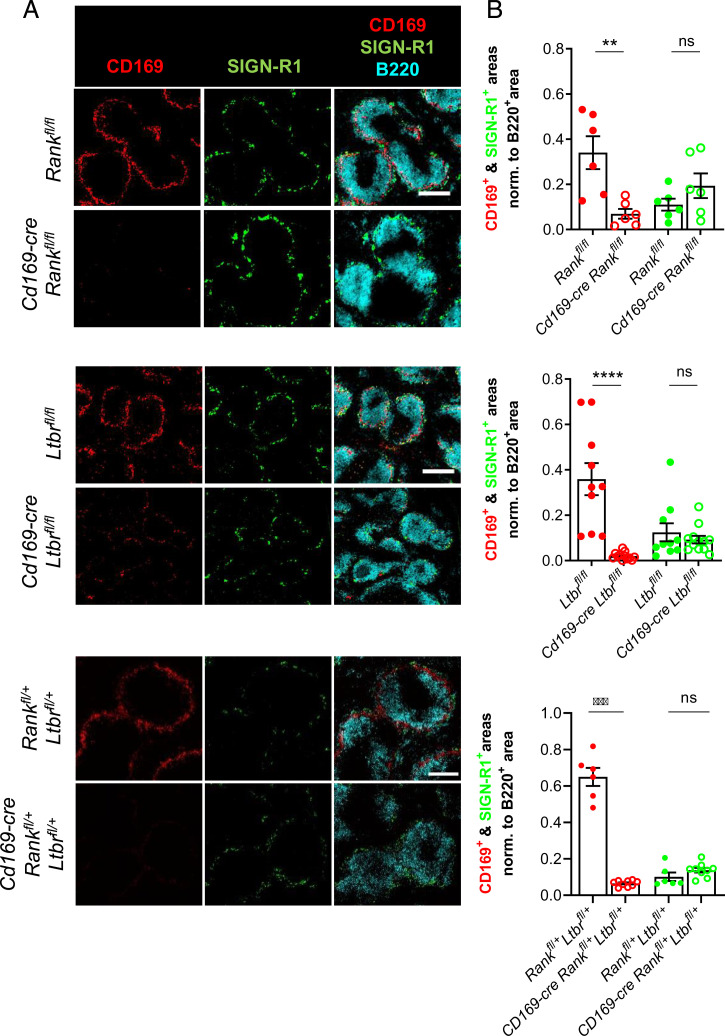
Loss of CD169^+^ MMMs through RANK and LTβR conditional deficiency. (*A*) Wide-field immunofluorescence microscopic images for CD169, SIGN-R1, and B220 of spleen sections from *Cd169-cre Rank^fl/fl^*, *Cd169-cre Ltbr^fl/fl^*, and *Cd169-cre Rank^fl/+^ Ltbr^fl/+^* with their respective littermate controls. (*B*) The graphs show the mean with individual data points of CD169^+^ and SIGN-R1^+^ areas in spleen sections normalized to the B220^+^ area. Statistical significance (Mann-Whitney test); ****P < 0.0001, ***P < 0.001, **P < 0.005; ns, not significant; error bar, SEM (scale bar, 200 μm).

To extend these observations to other lymphoid organs, we studied the spleen. It comprises CD169^+^ and SIGN-R1^+^ macrophages in the marginal zone (MZ), termed marginal metallophilic macrophages (MMMs) and MZ macrophages (MZMs), respectively. The proximity between CD169^+^ macrophages, MRCs, and B cells (25) is feature shared between the MZ and the LN subcapsular sinus. We therefore investigated a role of RANK and LTβR in the differentiation of MMMs. Immunofluorescence of spleen sections from *Cd169-cre Rank^fl/fl^*, *Cd169-cre Ltbr^fl/fl^*, and *Cd169-cre Rank^fl/+^ Ltbr^fl/+^*mice and their control littermates showed an almost complete absence of CD169 staining compared to littermate controls (Fig. 3 *A* and *B*). Yet, the staining for SIGN-R1 remained unchanged. The quantification of B220^+^ B cells and B cell follicles as well as expression of macrophage receptor with a collagenous structure (MARCO) was also similar between the genetically modified mice and their controls (*SI Appendix*, Fig. S3 *A*–*C*). In light of the close functional relationship between MMMs and MZ B cells (26), we next determined whether their loss provoked changes in the MZ B cell compartment. To visualize these B cells, the mice received an intravenous injection of anti-CD21/35 monoclonal antibody coupled to PE shortly before sacrifice (27). Immunofluorescence showed that the labeled MZ B cells were present and normally positioned (*SI Appendix*, Fig. S4*A*). Flow cytometry corroborated the normal numbers of CD21^+^ IgM^+^ IgG^−^ MZ B cells (*SI Appendix*, Fig. S4*B*) (4). To assess MZ B cell function, mice received 4-hydroxy-3-nitrophenylacetyl (NP)-Ficoll intravenously, and the NP-directed T cell–independent type II response was assessed. The capacity to raise an IgM immune response was not impaired in *Cd169-cre Ltbr^fl/fl^* mice (*SI Appendix*, Fig. S4*C*). These results support the conclusion that both the RANK and LTβR signaling pathways are required for MMM differentiation without negatively affecting other MZ cell populations.

### RANKL from Marginal Stromal Cell Supports MMM Differentiation.

**Fig. 4.**
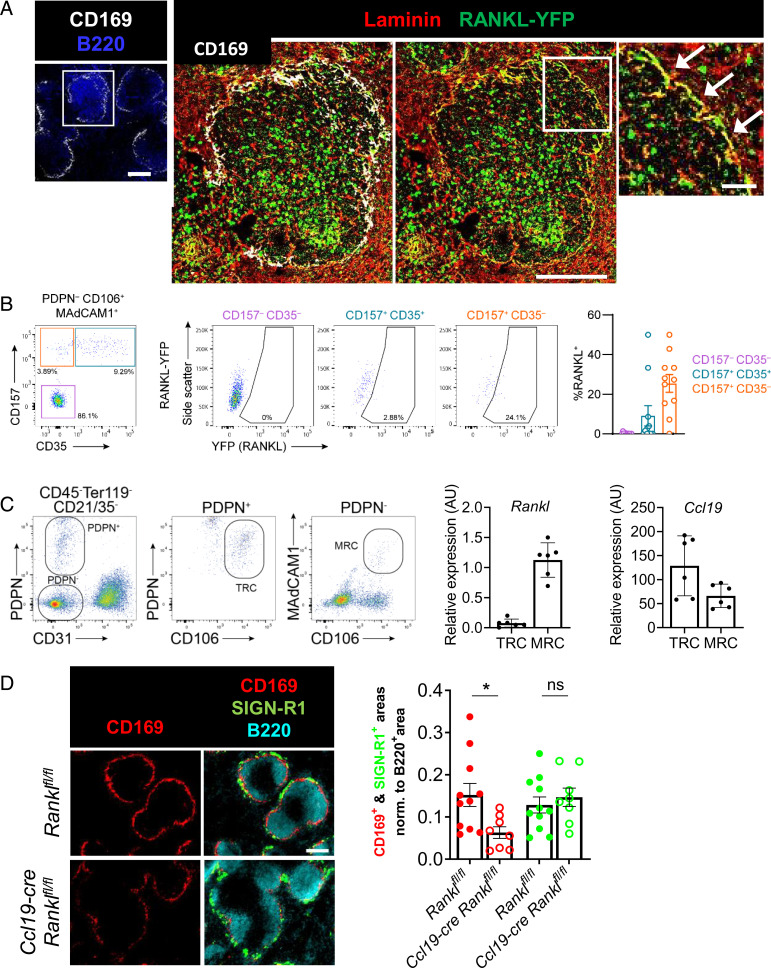
Splenic marginal reticular cells are a local source of RANKL for MMM differentiation. (*A*) Confocal microscopic images of spleen sections of RANKL reporter mice (RANKL-YFP) stained for YFP, CD169, B220, and laminin. The inset shows a higher magnification of RANKL-YFP–expressing laminin^+^ cells of the MZ. (*B*) Flow cytometry gating strategy to identify RANKL-YFP–expressing cells among the CD31^−^ Pdpn^−^ CD106/VCAM-1^+^ MAdCAM-1^+^ stromal cells. Cells with high YFP expression were found within the CD157^+^ CD35^−^ population. The graph summarizes the percentage of RANKL-YFP^+^ among the indicated cell types. Data points represent independent analyses, *n* = 11 from three independent experiments. (*C*) Gating strategy to sort TRCs and MRCs. The relative transcriptional levels of *Rankl* and *Ccl19* were assessed by RT-qPCR of the sorted cells. The data are from four independent experiments. (*D*) Wide-field immunofluorescence microscopic images of CD169, SIGN-R1, and B220 in *Ccl19-cre Rankl^fl/fl^* and *Rankl^fl/fl^* mice. Graph depicts the mean area of each macrophage marker with individual data points normalized to the B220^+^ area. Statistical significance (Mann-Whitney test); *P < 0.05; ns, not significant; error bar, SEM [scale bars, 100 μm (*A*, *Left* and *Middle*), 20 μm (*A*, *Right*), and 200 μm (*D*)].

The source of LTαβ in lymphatic organs under steady-state conditions is principally attributed to CXCL13-activated B cells (28). While RANKL is expressed by the MRCs of LNs (15, 25), the precise cellular source of RANKL in the spleen remains elusive (25). To overcome this difficulty, we generated *Rankl* knock-in reporter mice, in which an eYFP (enhanced yellow fluorescent protein) cassette replaced the stop codon at the *Rankl* locus (*SI Appendix*, Fig. S5*A*). Immunofluorescence analysis of spleen sections showed YFP expression in cells of the MZ, coexpressing the matrix protein laminin that is characteristic of the fibroblastic reticular cell network (Fig. 4*A*) (25). YFP^+^ stromal cells were also seen in the LN subcapsular area (*SI Appendix*, Fig. S6*A*). In addition, RANKL-YFP^+^ cells were scattered within the white and red pulp of the spleen and throughout the LN, suggestive of RANKL expression by hematopoietic cells (Fig. 4*A*; *SI Appendix*, Fig. S6*A*). We next used flow cytometry to assess RANKL expression by stromal and hematopoietic cells. Splenic stromal cells were identified among live CD45^−^ Ter-119^−^ CD31^−^ Pdpn^−^ cells, and the gating strategy was adjusted to identify MRCs as CD106^+^ MAdCAM-1^+^ CD157^+^ CD35^−^ cells (29). A high proportion of YFP^+^ cells was reproducibly found within this stromal subset (Fig. 4*B*). There was no YFP signal in these populations of wild-type control mice (*SI Appendix*, Fig. S5 *B* and *C*). Likewise, we detected strong signals in Pdpn^+^ CD31^−^ CD157^+^ MAdCAM-1^+^ LN MRCs (*SI Appendix*, Fig. S6*B*). We next sorted from the spleen Pdpn^−^ CD31^−^ CD106^+^ MAdCAM-1^+^ cells comprising MRCs and Pdpn^+^ CD106^+^ stromal cells that include T cell zone fibroblastic reticular cells (TRCs) and performed RT-qPCR for *Rankl* and *Ccl19*. The MRCs, but not the TRCs, transcribed *Rankl*, while both populations expressed *Ccl19* mRNA (Fig. 4*C*). With regard to hematopoietic cells, we detected in spleen and LNs YFP signals in CD4^+^ T cells, NK1.1^+^ CD127^+^ cells, NK1.1^+^ TCRγδ^+^ T cells, and NK1.1^−^ TCRγδ^+^ T cells. There was no expression in NK cells, CD8^+^ T cells, B cells, or dendritic cells (*SI Appendix*, Fig. S7 *A*–*C*). We next addressed the question of whether RANKL expressed by splenic MRCs is required for MMM differentiation. In light of the coexpression of CCL19 and RANKL by MRCs and the validity of the *Ccl19-cre* mice to target the white-pulp stromal cells (29, 30), we analyzed mice with conditional deficiency of RANKL under control of the *Ccl19* promoter (*Ccl19-cre Rankl^fl/fl^*) (15). The spleens were imaged after immunofluorescence for CD169, SIGN-R1, and B220 and the signals quantified. There was a reduction of CD169^+^ staining, while that of SIGN-R1 remained unchanged (Fig. 4*D*). In addition, we analyzed the spleens of mice with unconditional *Rankl* deletion (31) and confirmed a significant decrement of CD169 (*SI Appendix*, Fig. S8 *A* and *B*). Taken together with the selective expression of RANKL-YFP, this highlights splenic MRCs as a local source of RANKL that participates in the differentiation of the CD169^+^ MMMs.

### Diminished Virus-Specific Cytotoxic T Lymphocyte Priming in *Cd169-cre Ltbr^fl/fl^* Mice.

**Fig. 5.**
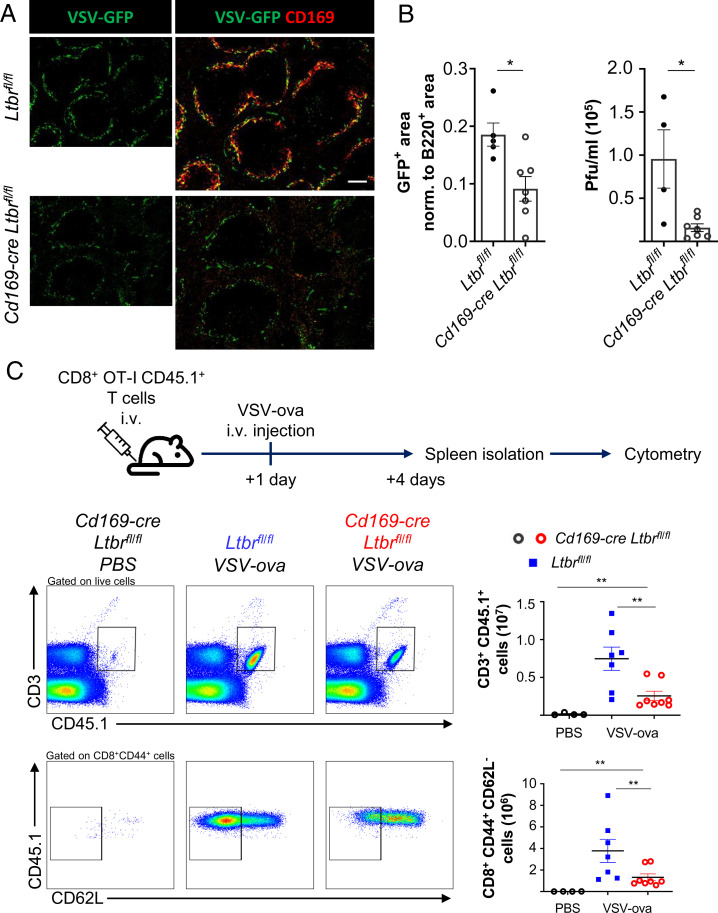
Impaired antiviral immunity in the absence of MMMs. (*A*) Wide-field immunofluorescence images for fluorescent VSV (VSV-GFP) and CD169 of spleens from *Cd169-cre Ltbr^fl/fl^* and *Ltbr^fl/fl^* mice. The graph depicts the mean area of GFP with individual data points normalized to the B220^+^ area. (*B*) The graph shows the quantification of the viral titer in the spleen of the indicated mice by measuring plaque-forming units. Shown are the mean with data points of individual mice. Statistical significance (Mann-Whitney test); *P < 0.05; error bar, SEM. (*C*) Experimental design for infecting mice with VSV-ova 24 h after bone marrow transfer of 5 × 10^6^ CD45.1 × OT-I mice. Gating strategy of flow cytometry analysis of ova-specific donor (CD45.1^+^ OT-I) T cells and their differentiation into cytotoxic CD8^+^ CD44^+^ CD62L^−^ T cells 3 d after intravenous infection of ova-expressing VSV. Graphs depict the numbers of CD3^+^ CD45.1^+^ T cells (*Top*) and of CD8^+^ CD44^+^ CD62L^−^ CD45.1^+^ T cells (*Bottom*). Shown are the mean values with data points of individual mice. Statistical significance (Mann-Whitney test); **P < 0.005; error bar, SEM (scale bar, 100 μm).

MMMs play an important role in immunity to blood-borne viruses such as vesicular stomatitis virus (VSV) by capturing virus for an efficient antiviral adaptive immune response (32). To assess the functional impact of the diminished numbers of MMMs in *Cd169-cre Ltbr^fl/fl^* mice, we infected the mice with VSV expressing green fluorescent protein (VSV-GFP) and determined GFP expression in spleen sections 6 h later. In agreement with the implication of MMMs in VSV infection (32), we saw a reduced expression of GFP in *Cd169-cre Ltbr^fl/fl^* mice compared to control animals (Fig. 5*A*). Immunostaining with CD169 confirmed infection of MMMs in the spleen of control mice (Fig. 5*A*). The remaining GFP signal likely originated from the MZMs that were preserved in the LTβR-deficient mice. There was also a diminished number of infectious viral particles in the spleens of the knock-out mice (Fig. 5*B*). To assess the antiviral immune response, we adoptively transferred ovalbumin-specific CD45.1^+^ CD8^+^ T cells from CD45.1 × OT-I transgenic mice to CD45.2 *Cd169-cre Ltbr^fl/fl^* and control mice before infection with VSV expressing the ovalbumin (ova) protein. Three days later, the ova-specific T cell response was measured in the spleen by flow cytometry. In comparison to uninfected mice, infected *Ltbr^fl/fl^* control mice showed a prominent CD45.1^+^ donor T cell population. However, their expansion in response to VSV-ova was greatly diminished in the *Cd169-cre Ltbr^fl/fl^* animals. In addition, we observed a clear reduction in the number of CD62L^−^ CD44^+^ effector CD8^+^ T cells (Fig. 5*C*). These data provide evidence that the conditional ablation of LTβR in MMMs leads to a reduced VSV replication and defects in the antiviral adaptive immune response.

## Discussion

Resident macrophages are responsive to environmental cues to shape their tissue-specific identity and functionality. We show here that CD169^+^ macrophages were directly responsive to RANKL and LTαβ signaling, since absent or reduced expression of their respective receptors resulted in a decline of their numbers. In LNs, they were replaced by myeloid cells lacking CD169 that could express SIGN-R1 and F4/80. Moreover, by studying compound heterozygous mice, we uncovered a collaborative activity of RANK and LTβR. Through the use of a RANKL reporter mouse, we identified the splenic MRCs as the principal RANKL-producing stromal cell. Mice with *Ccl19-cre*–mediated conditional ablation of *Rankl* revealed a significant impact of stromal RANKL on MMM formation. Lack of CD169^+^ macrophages resulted in defects in lymph-borne antigen uptake and a reduction in the CD8 T cell response to blood-borne virus.

The absence of B cells or the untargeted disruption of LTβR signaling results in the loss of SSMs and MMMs and, in the LN, their replacement by SIGN-R1^+^ cells (3–6, 8). These data were interpreted as evidence that LTαβ produced by CXCL13-activated follicular B cells is required for the differentiation of CD169^+^ macrophages. However, confirmation for LTβR signaling in CD169^+^ macrophages has so far been missing. The conditional deficiency of LTβR in *Cd169-cre* mice demonstrated that both SSMs and MMMs are dependent on the expression of LTβR. Also, the conditional deficiency of RANK resulted in loss of SSMs and MMMs. Remarkably, haploinsufficiency of both RANK and LTβR receptors sufficed to ablate the numbers of SSMs and MMMs to levels seen with complete gene deletion, validating that both receptors contribute equally to SSM and MMM differentiation. Interestingly, the unconditional ablation of one *Rankl* allele sporadically disrupts LN development, supporting the notion of a RANK–RANKL signaling threshold (33). Therefore, both RANK and LTβR signaling axes are required for the differentiation of the LN and the splenic CD169^+^ subsets (*SI Appendix*, Fig. S9).

SSMs were replaced by CD11b^+^ cells with some reminiscence of medullary sinus macrophages. In view of the role that SSMs play in the defense against infectious organisms and in the transfer of antigen to B cells, these changes may have important consequences. Indeed, similar to the absence of CD169^+^ SSMs owing to the loss of stromal RANKL (15), insufficient receptor signaling in compound heterozygous mice resulted in reduced antigen transport from lymph to the B cell follicle, a prerequisite for an efficient humoral response (7). The strong expression of RANKL by MRCs throughout the LN subcapsular zone may therefore be a means to assure the presence of SSMs in newly formed or secondary B cell follicles. Bone marrow transfer experiments in nonirradiated neonatal mice revealed a low but detectable replacement of LN macrophages by bone marrow–derived precursors. The significantly increased recruitment of SSMs in the LTβR-deficient mice suggest that the SIGN-R1^+^ CD169^−^ SSMs of the LNs may derive from sources other than embryonic tissues such as circulatory monocytes.

In the spleen, RANKL is poorly detectable by immunohistology or flow cytometry (25). The underlying reason is probably its low expression since the gene does not stand out as a principal cell marker although transcripts are detected in the transcriptome of splenic stromal cells (29). Indeed, we found low levels of *Rankl* expression in sorted MRCs and therefore used a YFP reporter mouse to better assess its cell source. The splenic MRCs were identified as the principal stromal cell type expressing RANKL-YFP, although fluorescence intensity was low and not all MRCs expressed the reporter protein. Likewise, RANKL-YFP was detected in LN MRCs. In line with this result and supporting a role of MRC RANKL in MMM differentiation, the conditional deficiency of stromal RANKL through *Ccl19*-mediated ablation led to a reduction in MMMs. The reporter mouse also revealed RANKL-YFP expression in CD4^+^ T cells, ILC1-like cells, and γδ T cells, confirming and refining transcriptomic datasets (https://www.immgen.org). In light of the incomplete loss of MMMs in *Ccl19-cre Rankl^fl/fl^* mice, it suggests that RANKL from hematopoietic sources may complement MRCs for MMM differentiation. In the spleen, we did not detect the replacement of MMMs by SIGN-R1^+^- or MARCO^+^-expressing myeloid cells. The reason for this difference is currently unclear but may suggest that the cell dynamics and/or the niche formation diverge between the two organs.

RANK, which is primarily known to stimulate the canonical NF-κB pathway, may complement the noncanonical pathway triggered by LTβR, similar to TNFR1-LTβR signaling (34). On the other hand, the two NF-κB pathways can also negatively interfere, leading to suppression of LTβR signaling (35). Although many myeloid cells coexpress LTβR and RANK (10, 36, 37), so far, the functional impact for parallel engagement of these two TNFSF receptors in other myeloid cells awaits further investigations and may shed light on their recruitment and differentiation processes.

Because the specific absence of MMMs in mice with *Cd169-cre*–mediated LTβR or RANK deficiency is not matched by other mouse models in which other cell types such as B cells or macrophages are affected (3–6, 8, 38–40), we assessed the MZ B cell compartment in *Cd169-cre Ltbr^fl/fl^* mice. There was no negative influence on MZ B cell formation, their positioning, or their function, indicating that MMMs are dispensable for this splenic B cell subset. Instead, and in line with the role of CD169^+^ macrophages in the capture and the immune response against viruses (32, 41, 42), we found a reduced VSV infection in the spleen and an attenuated virus-specific CD8^+^ T cell output. The importance of CD169^+^ macrophages in stimulating the cytotoxic T cell immune response (43, 44) has spurred investigation into their targeting for cancer therapy (45, 46). Although the dependence of CD169^+^ macrophages on intact RANK and LTβR axes remains to be confirmed in humans, the findings presented here are likely to raise interest, particularly in this therapeutic context.

## Materials and Methods

### Mouse Strains.

C57BL/6J (Charles River Laboratories), *Cd169-cre* (47), *Rank^fl/fl^* (48), *Ltbr^fl/fl^* (12), *Ccl19-cre* (14), *Rankl^fl/fl^* (49), *Rankl^−/−^* (31), CD45.1 (B6.SJL-Ptprca Pepcb/BoyJ), OT-I [C57BL/6-Tg(TcraTcrb)1100Mjb/J], and RANKL-YFP knock-in [B6/J-Tnfsf11(em[P2AEYFP])Ltk] mice were kept in specific pathogen–free conditions. Mice of both sexes were used at adult age (6 to 12 wk), except when indicated, and littermates were used as controls. All experiments were carried out in conformity to the animal bioethics legislation approved by and according to national guidelines of the Comité Régional d’Ethique en Matière d’Expérimentation Animale de Strasbourg.

### Generation of RANKL-YFP Reporter Mice.

RANKL-YFP knock-in reporter mice were generated using CRISPR/Cas9-mediated recombination. Double-strand DNA break was generated using guide RNA targeting the region near the stop codon of the *Tnfsf11* gene. Homology-directed repair donor was synthesized as a 1.1-kb single-stranded DNA megamer that contains left and right homology arms and GSG-P2A-EYFP cassette with the last 30 bases of exon 5 of *Tnfsf11* gene reworded without stop codon. P2A (ATNFSLLKOAGDVEENPGP) is a self-cleaving peptide derived from porcine teschovirus-1. Successful genomic recombination was screened using two pairs of primers: *Rankl* left (forward 5′-GCTGATGGTGTATGTCGT-3′, reverse 5′-TCAGGTAGTGGTTGTCGG-3′) and *Rankl* right (forward 5′-ACGACGGCAACTACAAGA-3′, reverse 5′-ACCCCCTTCCATAGCTCA-3′). Founders were genotyped by PCR using the following primers: 5′-CCGAGCTGGTGAAGAAATTAG-3′, 5′-ACGACGGCAACTACAAGA-3′, and 5′-ACCCCCTTCCATAGCTCA-3′. Mice homozygous for the knock-in allele were used for experiments.

### Immunofluorescence Microscopy.

LNs and spleens were freshly harvested, either fixed with 4% paraformaldehyde (PFA) solution in phosphate-buffered saline (PBS) overnight and/or directly embedded in the matrix for cryo-sectioning (Cell Path OCT Embedding Matrix), and frozen on dry ice. Then, 8- to 10-μm sections were prepared on the Cryostat (Leica CM3050 S). Non–PFA-fixed samples were soaked for 20 min in cold acetone for fixation. For immunostaining, samples were blocked in 2% serum in PBS for 30 min at room temperature and then incubated with primary antibodies for 30 min or 1 h at room temperature or overnight at 4 °C. The following primary and secondary antibodies and streptavidin conjugates purchased from Abcam, BD Biosciences, eBioscience, BioLegend, BioXcell, Cosmo Bio Co Ltd., Thermo Fisher Scientific, Molecular Probes, Sigma-Aldrich, Rockland, and R&D Systems were used: CD11b (M1/70), B220/CD45R (RA3-6B2), CD169 (3D6-112 and MOMA-1), SIGN-R1 (22D1), MARCO (goat BAF2956), RANKL (IK22-5), F4/80 (BM8), YFP (Rockland 600–101-215), pan-laminin (LSL LB-1013), alpha-smooth muscle actin (1A3), CD35 (8C12), goat anti-GFP, anti-goat IgG (A11055), anti-rabbit IgG (A32790), and streptavidin conjugates. DAPI (Sigma-Aldrich) was used to stain nuclei. Wide-field imaging was performed on the spinning disk microscope Zeiss Axio Observer Z1 and confocal imaging on the LSM780 (Carl Zeiss) and the SP5 (Leica, upright microscope DM6000CS). Image processing was performed with the open-source ImageJ software. To quantify cell-specific marker expression, a threshold cutoff was set for each staining, and the area of the selected mask was determined. Data were compiled and analyzed on GraphPad Prism 8.

### Single Cell Preparation.

LNs were cut into small pieces and digested with 1 mg/mL collagenase D (Roche), 1 mg/mL dispase II (Roche), and 0.1 mg/mL DNase I (Roche) in cell culture medium containing 2% fetal calf serum (FCS) for 1 h at 37 °C under agitation. Spleens were digested in 2 mg/mL collagenase D (Roche), 0.8 mg/mL dispase II (Roche), and 0.1 mg/mL DNase I (Roche). Otherwise, spleens were weighed and directly grinded in a glass putter with PBS. Samples were filtered through 40- to 70-μm cell strainer to exclude aggregates. If required, the red blood cells were lysed with ammonium-chloride-potassium buffer. The single-cell suspension was washed and prepared in PBS containing 10% FCS and 2.5 mM ethylenediaminetetraacetic acid. Splenic stromal cells were prepared as previously described (29).

### Flow Cytometry and Cell Sorting.

Single cells were split into 10^6^ cells/well (V-bottom 96-well plate) and blocked with 5% normal rat serum if required and FcR blocking reagent (Miltenyi Biotec) in PBS for 20 min at 4 °C and surface stained for 30 min at 4 °C with primary antibodies purchased from BD Biosciences, eBioscience, BioLegend, and BioXcell: CD3e (145-2C11), CD8a (53-6.7), CD11b (M1/70), CD11c (HL3), CD21/35 (8D9), CD31 (390), CD44 (IM7), CD45 (30F11), CD45.1 (A20), B220/CD45R (RA3-6B2), CD62L (MEL-14), CD106 (429), CD169 (3D6-112), SIGN-R1 (22D1), I-A/I-E (M5/114.15.2), F4/80 (BM8), Ter-119 (TER-119), podoplanin (8.1.1), MAdCAM-1 (MECA-367), CD21/CD35 (8D9), IgD (11-26c), and IgM (R6-60.2). For biotinylated antibodies, streptavidin conjugates (Molecular Probes, BioLegend) were used for 20 min at 4 °C. DAPI or propidium iodide was used to exclude dead cells. Flow cytometry was performed on a Gallios (Beckman-Coulter) or an LSRFortessa (BD Biosciences) and cell sorting on a FACS Aria III (Becton Dickinson). Data were analyzed on FlowJo software 10.1 (Treestar), compiled, and statistically analyzed on GraphPad Prism 8.

### Quantitative Real-Time PCR.

Total RNA was extracted from sorted samples using RNeasy Plus Micro Kit (Qiagen) and converted to complementary DNA (cDNA) using the High Capacity cDNA Reverse Transcription Kit (Thermo Fisher Scientific) according to the manufacturer’s instructions. Genes of interest were preamplified from cDNA using TaqMan PreAmp Master Mix (Thermo Fisher Scientific), and samples were analyzed by real-time qPCR using Fast SYBR Green Master Mix (Fischer Scientific). Cycle-threshold values were determined for genes individually, and gene expression was normalized to the housekeeping gene *Hprt* (ΔCt) and presented as 2 − ΔCt [arbitrary units (AU)]. The following probes were used: *Hprt* forward primer (5′ → 3′) TCAGTCAACGGGGGACATAAA, Hprt reverse primer (3′ → 5′) GGGGCTGTACTGCTTAACCAG, *Ccl19* forward primer (5′ → 3′) GGGGTGCTAATGATGCGGAA, *Ccl19* reverse primer (3′ → 5′) CCTTAGTGTGGTGAACACAACA, *Rankl* forward primer (5′ → 3′) CAGCCATTTGCACACCTCAC, and *Rankl* reverse primer (3′ → 5′) GTCTGTAGGTACGCTTCCCG.

### Infection and Viral Replication.

Mice were infected intravenously with 2 × 10^7^ plaque-forming units (pfu) of recombinant VSV-GFP, and spleens were harvested 6 h later. To analyze viral capture by microscopy, the spleen halves were directly fixed with 4% PFA in PBS overnight. To analyze the viral replication, the other halves were separately crushed in cell culture medium/FCS 10% with an Omni Tissuemaster 125 homogenizer (Dutscher). The homogenized samples were filtered to remove the cellular debris, and supernatants were transferred to new tubes. Virus infectivity for each sample was determined by plaque formation on monolayers of Vero R cells seeded in 24-well plates. Cells were infected for 1 h with supernatant from spleen homogenates prepared in 10-fold dilution cascades. Cells were cultured in 2.5% carboxymethyl cellulose diluted in DMEM/FCS 10% for 72 h at 37 °C in a humidified atmosphere of 5% CO_2_. After medium removal, cells were fixed with 4% formaldehyde for 20 min and stained with 1× crystal violet solution [2% crystal violet (Sigma-Aldrich), 20% ethanol, 4% formaldehyde]. The VSV-GFP titer determination was performed by plate counting.

### Adoptive Transfer and Immunization.

Mice received intravenous ( i.v.) injection of 1.5 × 10^6^ CD8^+^ T cells isolated from spleens and peripheral LNs of OT-I × CD45.1 mice using CD8a^+^ T Cell Isolation Kit (Miltenyi Biotec). After 24 h, recipients were infected by i.v. injection of 5 × 10^5^ pfu of VSV-ova. After 3 d, spleens were harvested, and T cells were extracted and analyzed by flow cytometry. For bone marrow transfers, 2-d–old mice received intraperitoneal (i.p.) and i.v. injection of 5 × 10^6^ bone marrow cells isolated from 4-wk–old CD45.1 mice. After 4 wk, mice were killed and the inguinal and brachial LNs collected and analyzed.

### Immunization and ELISA.

Mice were immunized with PE–immune complexes by injecting i.p. 2 mg of rabbit IgG (Rockland) 16 h before intrafootpad and intra-auricular administration of 10 μg of PE (Thermo Fisher Scientific). Mice were killed 8 h later and popliteal and auricular LNs imaged. The T cell–independent type 2 immune response was elicited by injecting intravenously 25 μg of NP_49_-AECM-Ficoll (4-hydroxy-3-nitrophenylacetyl conjugated to aminoethylcarboxymethyl-Ficoll; Biosearch Technologies). After 7 d, mice were bled, and the anti-IgM was quantified by ELISA. After coating ELISA plates with 4-hydroxy-3-nitrophenyl acetyl (NP)_5_-BSA (10 μg/mL overnight, 4 °C; Biosearch Technologies) and blocking with 1% BSA, the plates were incubated with serial dilutions of mouse serum for 2 h at 37 °C. After second washing, bound IgM was detected with horseradish peroxidase–conjugated goat anti-murine IgM (Bethyl Laboratories). The reaction was stopped by addition of 2 N HCl and the optical density measured at 450 nm. The concentration of total serum Ig was evaluated by comparison with a standard curve (Bethyl Laboratories). NP-specific relative Ig titers were calculated by normalizing titers at a specific optical density using the mean titer of dilutions encompassing this optical density.

## Supplementary Material

Supplementary File

## Data Availability

All study data are included in the article and/or *SI Appendix*.
