# Supplementary material for: CD169+ macrophages in lymph node and spleen critically depend on dual RANK and LTbetaR signaling
Source: Proc Natl Acad Sci U S A. 2022 Jan 14;119(3):e2108540119. doi: 10.1073/pnas.2108540119 (PMC8784161; doi:10.1073/pnas.2108540119)
Supplement: Supplementary File [file pnas.2108540119.sapp.pdf]

Supplementary Information for

CD169^{+} macrophages in lymph node and spleen critically depend on dual RANK and LTbetaR signaling

Abdouramane Camara, Alice C. Lavanant, Jun Abe, Henri Lee Desforges, Yannick O. Alexandre, Erika Girardi, Zinaida Igamberdieva, Kenichi Asano, Masato Tanaka, Thomas Hehlgans, Klaus Pfeffer, Sébastien Pfeffer, Scott N. Mueller, Jens V. Stein, Christopher G. Mueller*

* Corresponding author: Christopher G. Mueller
Email: c.mueller@ibmc-cnrs.unistra.fr

This PDF file includes:

Figures S1 to S9

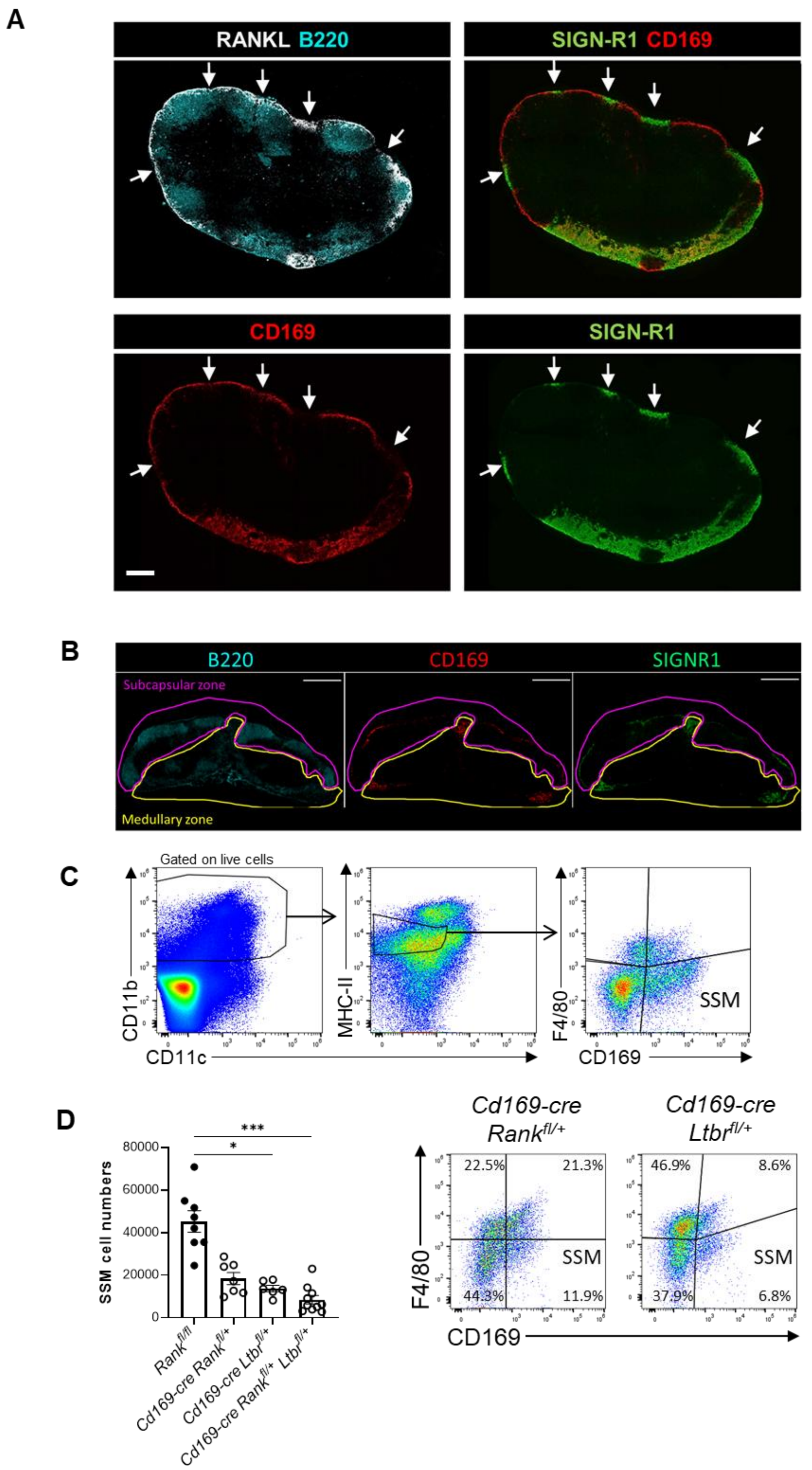

Fig. S1. (A) Wide field immunofluorescence microscopic images for RANKL, B220, CD169 and SIGN-R1 of inguinal LN sections from wild-type C57BL/6 mice. Arrows point to the inter B cell follicular areas. Scale bar = 200 µm. **(B)** Images from inguinal LNs illustrating the selection of the subcapsular and the medullary zones based on B220, CD169 and SIGN-R1 distribution. Scale bar = 500 µm. **(C)** Flow cytometry gating strategy to discern MSMs, SSMs and other LN macrophage populations. **(D)** Flow cytometry analysis to identify CD169^{+} F4/80^{-} SSMs of inguinal and brachial LNs pre-gated as live CD11b^{+} CD11c^{+} MHC-II^{Int} cells in mice of the indicated genotype. The proportion of the cells in the quadrants is indicated. The graph depicts the mean absolute SSM cell numbers with data points for each animal. Statistical significance (Mann-Whitney test); ns, not significant, **p < 0.002; error bar: SEM.

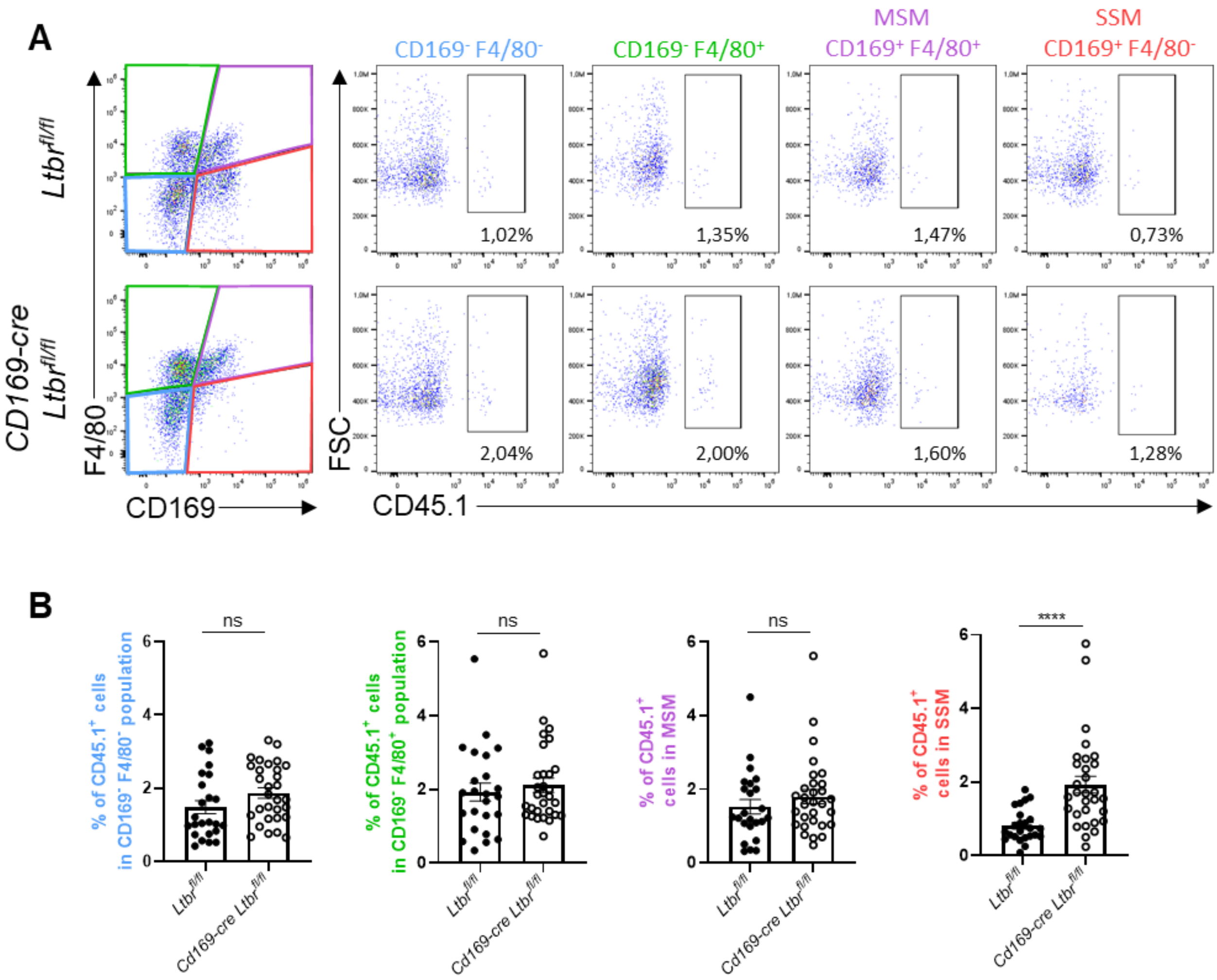

Fig. S2. **(A)** Identification of CD45.1⁺ bone marrow-derived LN macrophages 4 weeks after adoptive transfer to neonatal mice of the indicated genotypes. **(B)** The graphs summarize the mean proportion of CD45.1⁺ macrophage subsets, with data points for inguinal and brachial LNs. Statistical significance (Mann-Whitney test); ns, not significant, **p < 0.002; error bar: SEM.

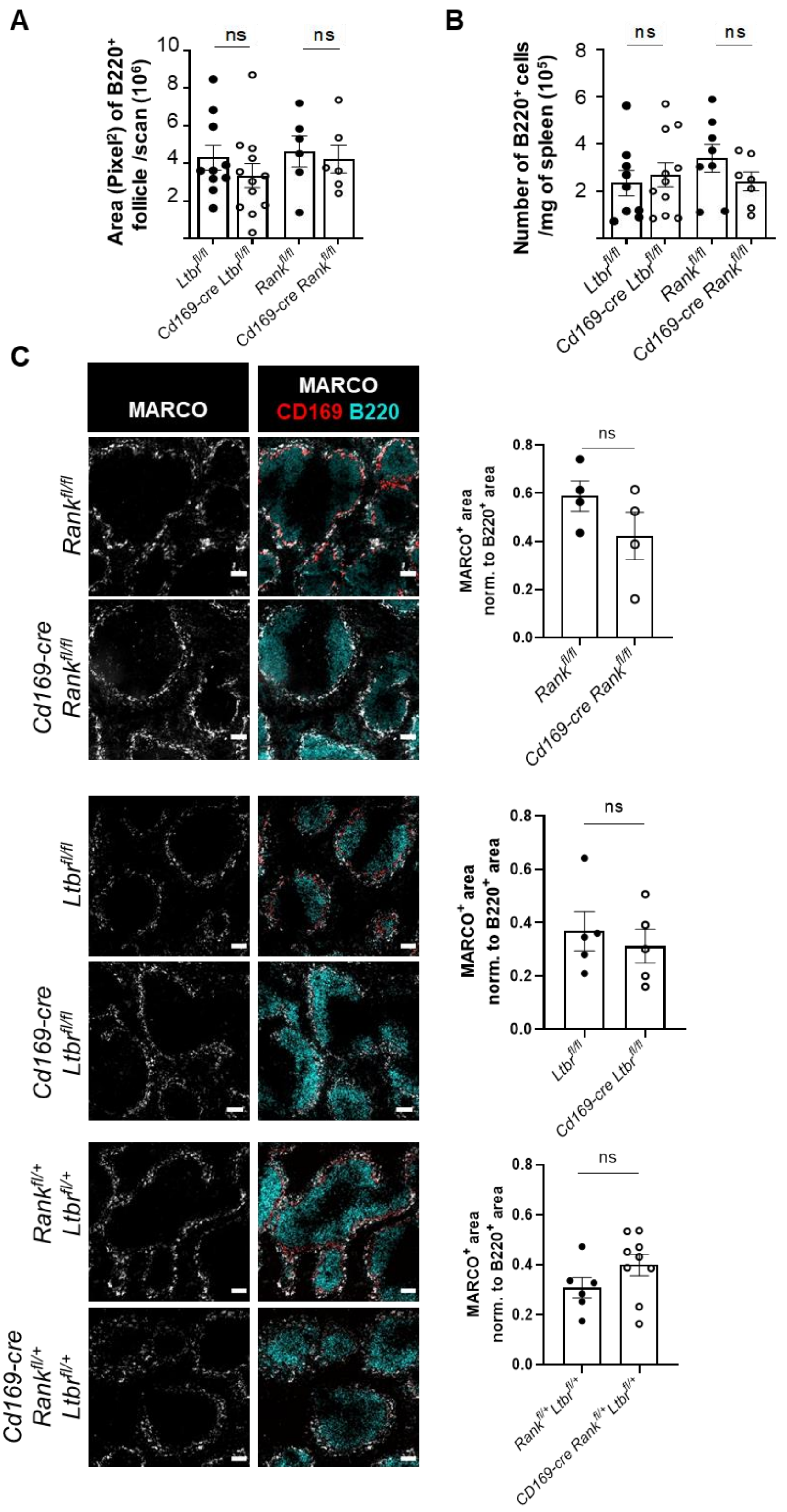

Fig. S3. (A) Spleens from mice with the indicated genotype were stained for B220. Graph depicts the area of B220⁺ follicles per image. (Mann-Whitney test); ns, not significant; error bar: SEM. **(B)** Flow cytometry quantification of B220⁺ cells in spleens from mice with the indicated genotypes. Graph depicts the number of B220⁺ cells per milligram of spleen. Data points represent individual mice. (Mann-Whitney test); ns, not significant; error bar: SEM. **(C)** Wide field immunofluorescence microscopic images for MARCO of spleen sections of the mice with the indicated genotypes (scale bars = 100 µm). Graphs depict the MARCO⁺ areas normalized to the B220⁺ areas. Data points represent the mean of images of different spleens. Statistical significance (Mann-Whitney test); ns, not significant; error bar: SEM.

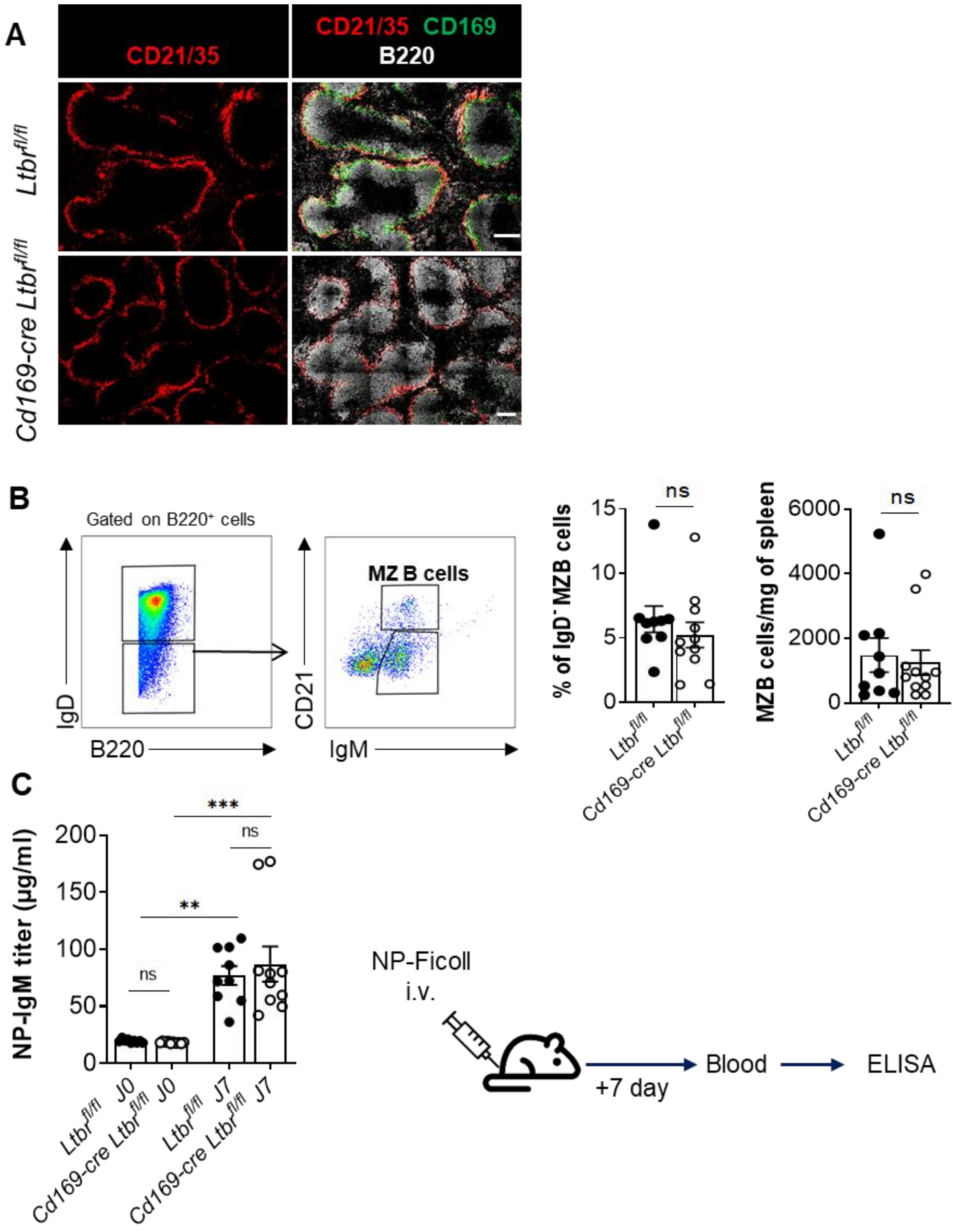

Fig. S4. (A) Spleens sections from mice having received intravenously 5 min before sacrifice anti-CD21/35-PE were imaged for PE-fluorescence without and with labeling for CD169 and B220. Scale bar = 100 µm. **(B)** Flow cytometry gating procedure to identify MZ B cells. Graphs show their relative and absolute numbers per milligram of spleen. Statistical significance (Mann-Whitney test); ns: not significant; error bar: SEM. **(C)** Anti-NP-specific IgM levels were measured in sera before and 7 days after i.v. injection of NP-Ficoll in the indicated mice. Graph shows the mean with data points for each mouse. Statistical significance (Kruskal-Wallis test); ns, not significant, **p < 0.002, ***p < 0.001; error bar: SEM.

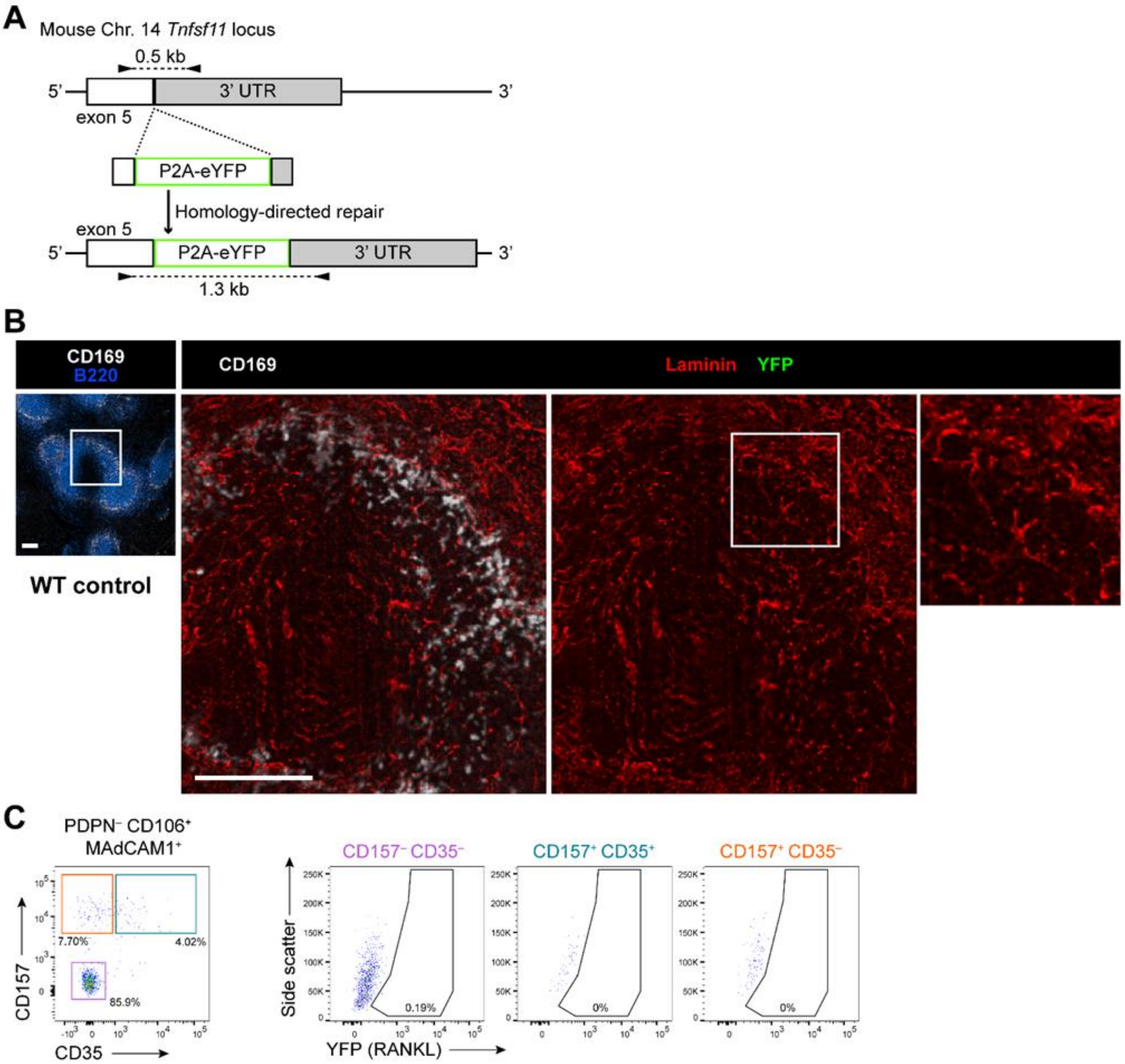

Fig. S5. (A) Schematic illustration of the genetic construction of the *Rankl* knock-in reporter mice, where a P2A-eYFP cassette replaced the stop codon at the *Rankl* locus. **(B)** Images of spleen section from WT littermate control stained for CD169, DAPI, laminin and RANKL-YFP (anti-GFP). Scale bar = 100 µm. **(C)** Flow cytometry of WT mice using the same setting as in *Rankl* knock-in reporter mice.

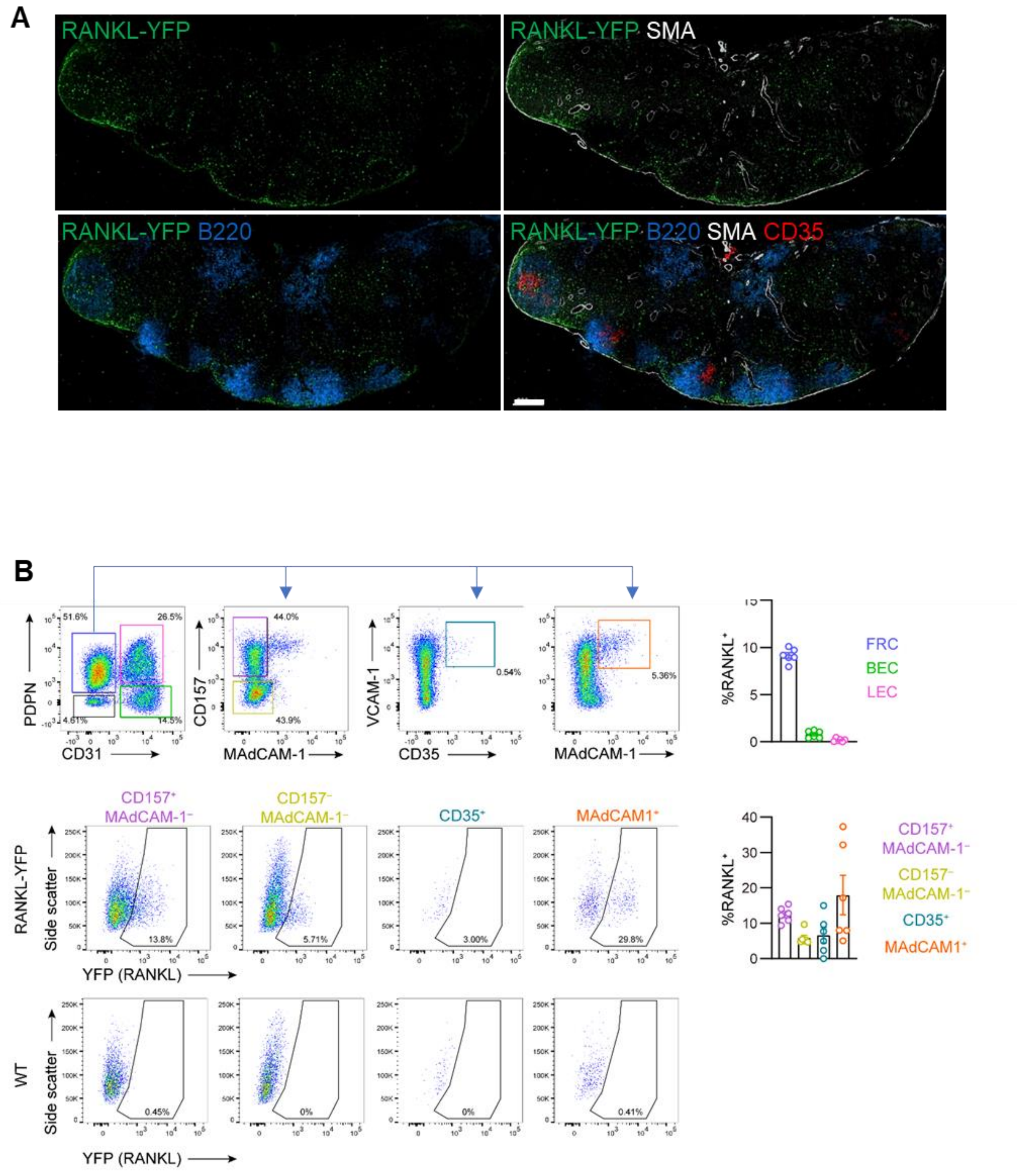

Fig. S6. (A) Images of LN sections from *Rankl* knock-in mice stained for RANKL-YFP (anti-GFP), B220, α-smooth muscle actin (SMA) and CD35. Scale bar = 200 µm. **(B)** Flow cytometry gating strategy to identify RANKL-YFP-expressing stromal cells. Top panel: among the mesenchymal stromal cells (FRCs), blood and lymphatic endothelial cells (BECs, LECs), bottom panel: among the different mesenchymal subsets including the MAdCAM-1^{+} MRCs. The graphs summarize the % RANKL-YFP^{+} among the indicated cell types. Data points represent independent analyses from three independent experiments.

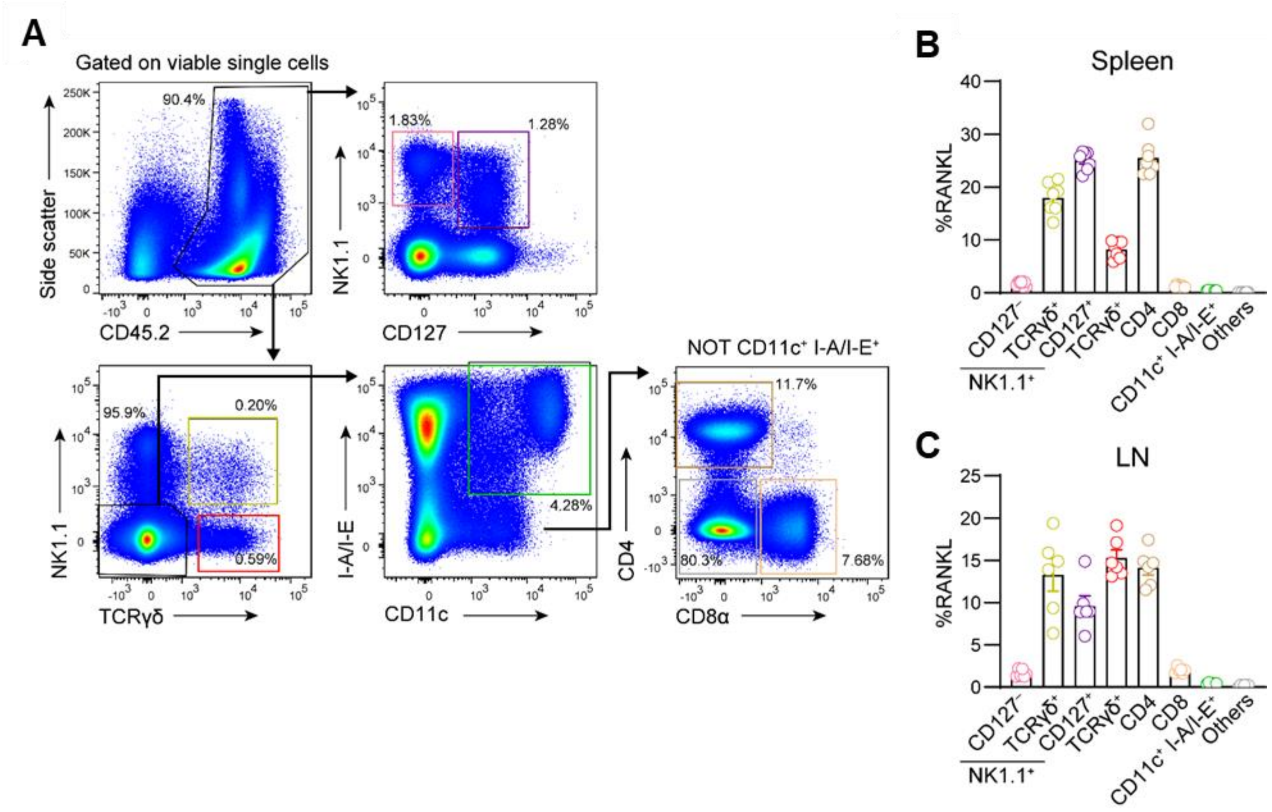

Fig. S7. (A) Flow cytometry gating strategy to identify RANKL-YFP-expressing cells among the $CD45^+$ hematopoietic cell subsets. The graph summarizes the % RANKL-YFP among the different cell types in **(B)** spleen and **(C)** in LNs. n=7 and 6 from two independent experiments.

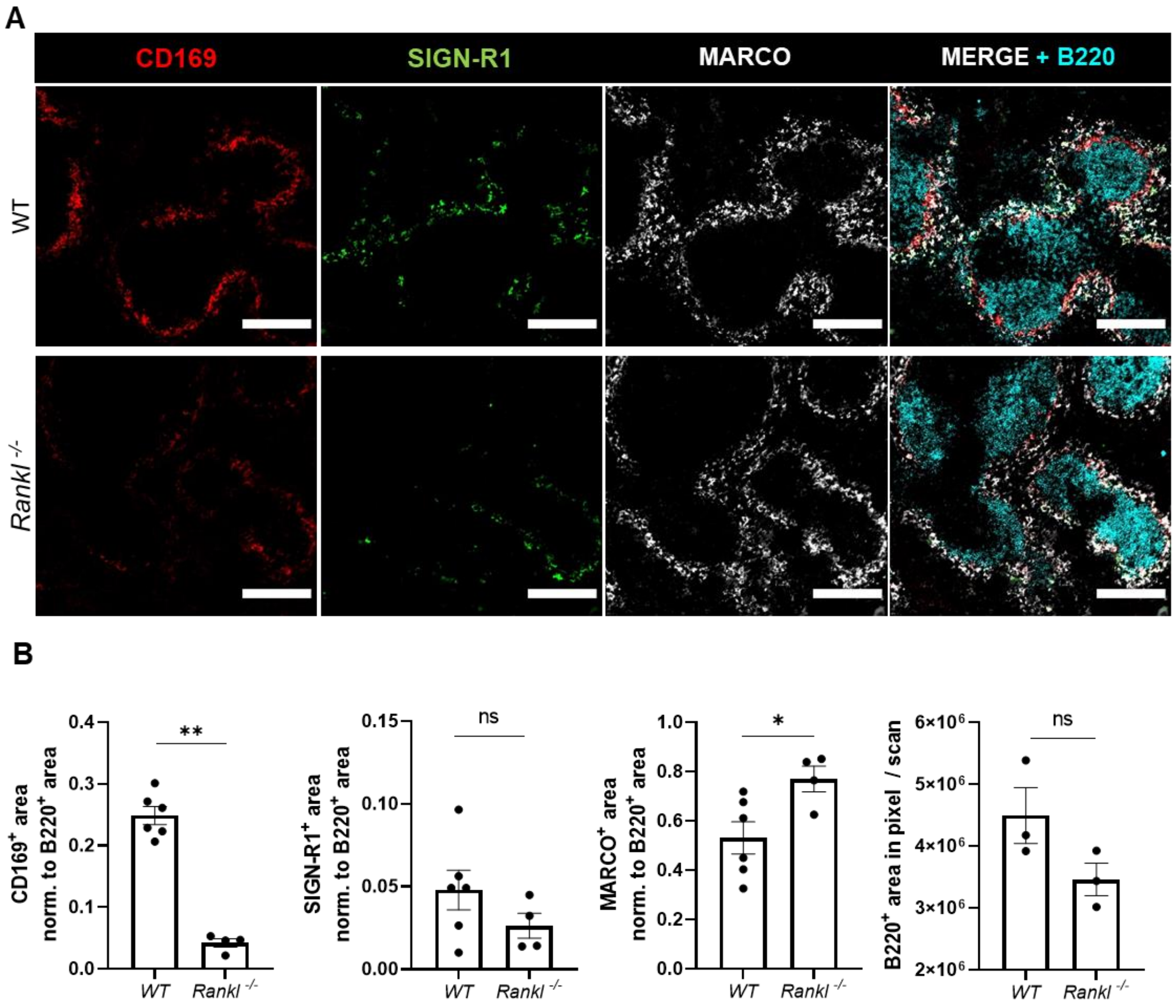

Fig. S8. (A) Spleens from unconditional *Rankl*-deficient or littermate WT mice were stained for CD169, SIGN-R1 MARCO and B220. Scale bar = 100 µm. **(B)** Graphs depict the quantification of CD169, SIGN-R1 and MARCO expression normalized to the B220+ area. Data points represent the mean of images of different spleens of two mice of each genotype. Statistical significance (Mann-Whitney test); ns, not significant, *p < 0.05,**p < 0.005; error bar: SEM.

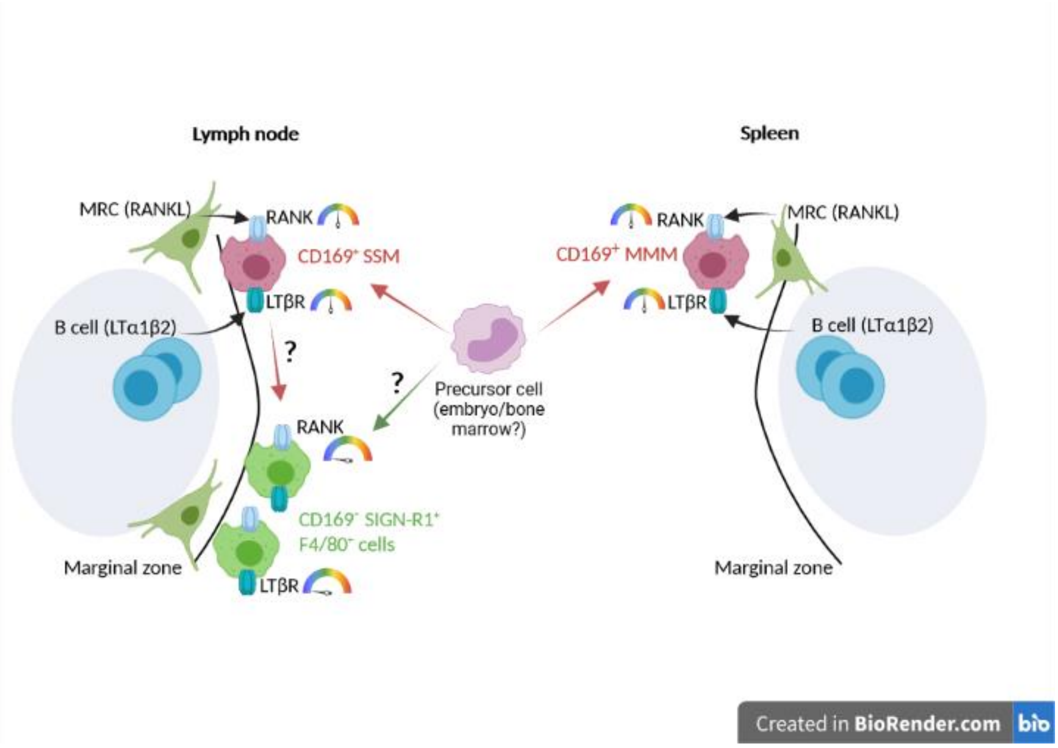

Fig. S9. Dependency of CD169$^+$ macrophages on RANK and LTβR receptors. Lymph node CD169$^+$ subcapsular sinus macrophages (SSMs) and spleen metallophilic macrophages (MMMs) require concurrent RANK and LTβR signalling for differentiation/maintenance. RANKL is constitutively expressed by the marginal reticular stromal cells (MRCs) and lymphotoxin LTα1β2 by activated B cells. In case of insufficient signal strength, the macrophages acquire new phenotypic and functional features either by conversion into a different subtype or by replacement from precursor cells.
